# Prevalence and Factors Associated with Driving Under the Influence of Alcohol in Brazil: An Analysis by Macroregion

**DOI:** 10.3390/ijerph17030767

**Published:** 2020-01-25

**Authors:** Rafael Alves Guimarães, Otaliba Libânio Morais Neto

**Affiliations:** Institute of Tropical Pathology and Public Health, Federal University of Goiás, Goiânia 74605-450, Brazil; otaliba.libanio@gmail.com

**Keywords:** drinking under the influence, alcohol, prevalence, Brazil

## Abstract

Objective: To analyze the prevalence and factors associated with driving under the influence of alcohol (DUIA) in Brazil, according to macroregion. Methods: A cross-sectional study was conducted using data from individuals aged 18 years or older who participated in the 2013 National Health Survey in Brazil. Subjects were selected by probabilistic sampling and interviewed through home visits. Prevalence of DUIA was estimated according to the number of drivers and/or motorcyclists who reported consuming alcohol in the previous 30 days (n = 9537). Poisson regression was used to analyze the factors associated with DUIA to Brazil and in each macroregion of the country. Results: The prevalence of DUIA was 27.5%, 29.4%, 29.6%, 22.9%, and 20.8% in the North, Northeast, Central-West, South, and Southeast macroregions, respectively. The overall prevalence of Brazil was 24.3%. In most macroregions, the main predictors of DUIA were male sex, high educational level, living in outside the capital or metropolitan regions (other regions), and binge drinking in the previous 30 days. Depression was a predictor in Brazil and two macroregions. Conclusion: A high prevalence of DUIA was observed in Brazil, especially in the Central-West, Northeast and, North macro-regions. Factors associated with DUIA can be incorporated to develop effective interventions to reduce this behavior in Brazil.

## 1. Introduction

Morbidity and mortality from road traffic injuries (RTIs) are a serious public health problem. World Health Organization estimates show that 50 million people are injured, and 1.35 million die annually as a result of RTIs (3700 deaths per day), accounting for approximately 2.5% of the world’s total deaths [1]. Additionally, it is expected that if preventative measures are not adopted and intensified, injuries resulting from RTIs will become the fifth leading cause of death worldwide by 2030 [2]. Approximately 93% of RTIs-related deaths occur in low- and middle-income countries, which contain 60% of the world’s registered vehicle fleet. Mortality rates are three times higher in low-income countries than in high-income countries [1].

Brazil has the third-highest number of RTIs-related deaths, with an estimated mortality rate of 19.7 per 100,000 inhabitants in 2016; this is exceeded only by India and China [1]. In 2013, RTIs were the cause of 170,805 admissions to the Brazilian public health system (*Sistema Único de Saúde*—in Portuguese), a rate of 85 admissions per 100,000 inhabitants, with the highest rates found in the Central-West and Northeast macroregions [3]. A mortality rate by RTIs of 21 deaths per 100,000 inhabitants was observed in Brazil in 2013, with the highest rate found in the Central-West macroregion [4]. Estimates show that RTIs-related deaths can result in costs of up to 6% of gross domestic product (GDP) [5]. In Brazil, RTIs-related costs in health services and productivity losses are also high [6], equivalent to 1.2% of GDP [7].

Driving under the influence of alcohol (DUIA) is one of the most critical risk factors for RTIs, and traffic-accident mortality [1,8,9]. Drivers with a blood alcohol concentration (BAC) of 0.02–0.05 g/dl have three times the risk of mortality, due to RTIs. This risk increases exponentially with an increase in BAC [10]. A meta-analysis showed that for every 10 g of alcohol in the blood, the chance of RTIs increased by 24% for accidents involving motor vehicles, and by 30% for non-motor-vehicle-related injury [8]. The WHO estimates that 20% of drivers involved in fatal accidents in high-income countries have high blood alcohol levels, and this estimate increases to 33–66% in low-income countries [11]. Alcohol, even in small quantities, increases the risk of involvement in and the potential severity of RTIs for both drivers and pedestrians because it alters individual’s ability to discriminate and control a vehicle, visual acuity, reaction time, information processing, and motor coordination [12]. Additionally, alcohol is associated with involvement in other risky behaviors, such as excessive speed and failure to use safety equipment [13,14].

Multiple factors are associated with DUIA. Community population-based studies show associations with male sex [15], binge drinking [15,16], regular alcohol use [15,17], non-use of seat belts, and previous RTIs [17]. Studies in sobriety checkpoints have shown associations between DUIA and male sex, single marital status [18], higher age [19,20,21], lower education level, binge drinking [20,21], and previous RTIs [19].

Developing countries, such as Brazil, have adopted policies to control DUIA, such as zero tolerance laws for BAC [22]. Despite these laws, studies have shown a high prevalence of DUIA in Brazil [19,21,23,24]. Boni et al. [21] investigated the prevalence of DUIA among drivers of cars and/or motorcycles in sobriety checkpoints and found a positive breathalyzer test frequency of 4.4% in the South and 8.1% in the Central-West macroregions. Using the same method, Campos et al. [19] verified a frequency of 15.9% in drivers from cities in the Southeast macroregion. A nationwide population-based study conducted in 2005–2006 showed a 6.4% prevalence of DUIA in the previous 12 months [24]. In 2013, data from the Surveillance System for Risk and Protective Factors for Chronic Diseases by Telephone Survey (*Sistema de Vigilância de Fatores de Risco e Proteção para Doenças Crônicas por Inquérito Telefônico* in Portuguese-Vigitel) conducted in Brazil showed a prevalence of 21.7% in the Southeast macroregion and 33.7% in the Central-West [23].

The National Health Survey (NHS) conducted in Brazil in 2013 [25] employed a direct household survey to investigate the prevalence of DUIA among drivers who had used alcohol within previous 30 days; it identified a DUIA prevalence of 24.3% [26]. However, the study did not consider regional differences, nor did it analyze the factors associated with DUIA in each macroregion. Brazil is a country of continental scale, with cultural and economic heterogeneity among its macroregions; these differences may lead to differences in the prevalence and determinants of DUIA [21]. Understanding region-specific risk factors associated with DUIA is crucial for designing effective interventions to prevent RTIs [27]. Therefore, this study aimed to analyze the prevalence and factors associated with DUIA in Brazil, according to macroregion.

## 2. Materials and Methods

### 2.1. Design, Sampling and Setting

This study analyzed data of drivers of cars and/or motorcyclists who had used alcohol within the previous 30 days interviewed during the NHS. The NHS, a cross-sectional, population-based, household study conducted in 2013 by the Brazilian Institute of Geography and Statistics and the Brazilian Ministry of Health, analyzed the health status and lifestyles of the Brazilian population [25]. During the survey, 60,202 participants aged 18 or over were interviewed through home visits, who answered data on self-reported diseases, access to and use of health services, and risk factors for noncommunicable diseases and injuries, such as RTIs [25].

The sampling strategy of the NHS has been described previously [25]. It involved complex sampling by groups conducted in three phases. The first phase selected primary sample units composed of census tracts of the respective participating municipalities. The second phase conducted a simple random selection of households from each primary sample unity as secondary sampling units. The third step selected random samples of adult residents from randomized households. Weighting factors were calculated for each of the three sampling units, accounting for the probabilities of selection and non-response rates [25].

The NHS was designed to present representativeness for Brazil, urban and rural areas, macroregions, Brazilian capitals and the rest of the metropolitan regions, justifying the use of three sampling stages [25]. In this study, we present the estimates of DUIA for Brazil, macroregions, states, and capitals and associated factors for Brazil and macroregions.

**Figure 1:**
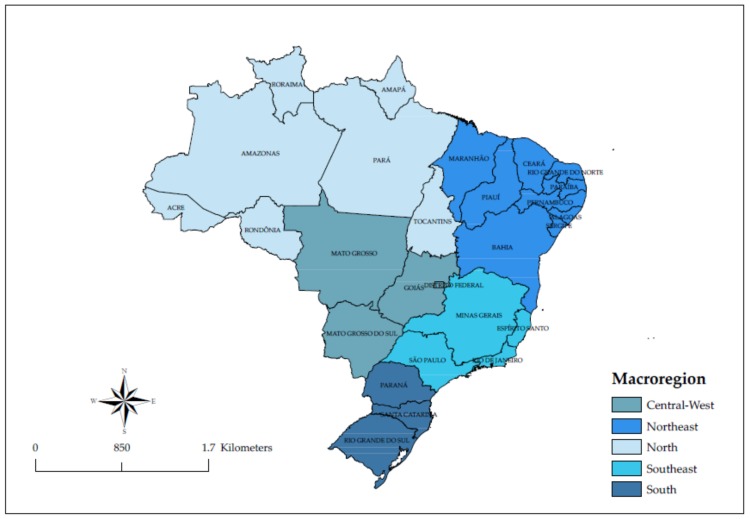
Geographic localization of Brazilian states and macroregions.

The setting of this study was Brazil and the macroregions. Brazil, a country with more than 200 million inhabitants, is divided into five geographic regions or macroregions. Each of them is comprised of three or more states and has its own population, social and economic characteristics. (Figure 1). The description of the macroregions and their states is as follows [28,29]:

(i) Macroregion Southeast: It is composed of the states of Rio de Janeiro, Sao Paulo, Espírito Santo and Minas Gerais. With an estimated population of 86.3 million inhabitants and has a population density of 87 inhabitants/km^2^. It is the richest in Brazil, contributing 53.2% of Brazil’s GDP;
(ii) Macroregion South: It is composed of the states of Rio Grande do Sul, Santa Catarina and Paraná. With an estimated population of 29.4 million inhabitants and has a population density of 50 inhabitants/km^2^. It is the second richest macroregion in Brazil, contributing 17% of Brazil’s GDP;
(iii) Macroregion Northeast: It is composed of the states of Maranhão, Rio Grande do Norte, Paraiba, Sergipe, Bahia, Alagoas, Pernambuco, Ceará and Piauí. With an estimated population of 56.9 million and a population density of 34 inhabitants/km^2^. It contributes 14.3% of Brazil’s GDP;
(iv) Macroregion Central-West: It is composed of the states of Goiás, Mato Grosso, Mato Grosso do Sul and Federal District that houses Brasilia, capital of Brazil. With an estimated population of 15.6 million inhabitants and has a population density of 10 inhabitants/km^2^. It contributes 10.1% of Brazil’s GDP;
(v) Macroregion North: It is composed of the states of Amazonas, Tocantins, Pará, Acre, Amapá, Roraima and Rondônia. With an estimated population of 17.7 million inhabitants and a population density of 4.6 inhabitants/km^2^. It contributes 5.4% of Brazil’s GDP.

### 2.2. Variables

#### 2.2.1. Dependent Variable

As previously described, the NHS collected data from 60,202 individuals from various health-related aspects [25]. As described in the research protocol published in previous reports [23,26], the target population for DUIA in NHS estimates was composed of drivers of cars and/or motorcycles who have consumed alcohol during the previous 30 days (n = 9527; 12.8% of the total NHS sample), this being the denominator for the DUIA prevalence calculation. This population answered the following question “On any of these days (previous 30 days) when you drank alcohol, did you drive right after drinking?” (no or yes) [23]. Thus, the dependent variable was DUIA within the previous 30 days, second self-report and the variable could assume only two values (0 = no or 1 = yes), for each participant.

#### 2.2.2. Independent Variables

The independent variables included: (i) sex: Female or male; (ii) age (years): 18–29, 30–39, 40–59, or ≥60 [30]; (iii) self-reported race/skin color: White, black, brown, or others (native Brazilian or Asian); (iv) education level: Illiterate or elementary school incomplete, elementary school complete or high school incomplete, high school complete or college school incomplete, or college school complete or above; (v) marital status: With partner or without partner [18]; (vi) residence area: Within the capital, within of metropolitan regions, or within other regions (residence outside the capital or metropolitan regions); (vii) age at start of alcohol use (years): ≥18 or <18 [15]; (viii) binge drinking in the previous 30 days: No or yes [15]; (ix) tobacco use in the previous 30 days: No or yes; and (ix) depression: No or yes. To account for variation in each macroregion, the state was also considered as an independent variable. To analyze predictors of DUIA for Brazil, macroregion was considered as an independent variable (Southeast, South, Central-West, Northeast, or North).

The categories for the race/skin color variable were defined based on the classification of this variable for the Brazilian population by the Brazilian Institute of Geography and Statistics [31]. Binge drinking was defined as consuming five or more alcohol units for men, or four or more for women, on a single occasion within the previous 30 days [32].

Depression was assessed using the Patient Health Questionnaire-9 (PHQ-9) scale, which has been validated for the general population of Brazil. This scale contains nine items, each addressing a symptom of depression according to the diagnostic criteria of the Diagnostic and Statistical Manual of Mental Disorders, Fourth Edition (DSM-IV). In the original scale, total PHQ-9 scores of 5–9, 10–14, 15–19, and 20 or more represent mild, moderate, moderately severe, and severe depression, respectively [33]. The PHQ-9 validation study in Brazil showed good validity for the diagnosis of major depression at the cutoff points of the scale equal to or greater than nine (≥9) in the general population, with a sensitivity of 77.5% (95.0% confidence interval [95.0% CI]: 61.5–89.2) and specificity of 86.7% (95.0% CI: 83.0–89.9) [34]. Thus, the present study defined depression as a PHQ-9 score of nine or greater.

### 2.3. Statistical Analysis

Data were analyzed using STATA version 15.0 (StataCorp LLC, College Station, TX, USA). First, a descriptive analysis of the variables was performed. Variables were presented as relative frequency and 95.0% CI. Cronbach’s alpha was used to verify the internal reliability of the PHQ-9, with acceptable reliability defined as a coefficient greater than 0.7 [35]. In this study, Cronbach’s alpha was 0.827, indicating good internal reliability. DUIA prevalence was estimated using a 95.0% CI for Brazil, each macroregion and, variable independent.

To verify the factors associated with DUIA, bivariate and multivariable analyses were performed using the “survey” package for complex samples. Variables with *p*-values below 0.20 and potential confounding sociodemographic factors (age and sex) were included in a Poisson regression model. Factors associated were analyzed according to Brazilian macroregion. Results of the bivariate analysis were presented as crude prevalence ratio (cPR) and 95.0% CI; results of the multivariable analysis were presented as adjusted prevalence ratio (aPR) and 95.0% CI. Variables with *p*-values below 0.05 after multivariable analysis were considered statistically significant.

### 2.4. Ethical Considerations

This study was approved by the National Committee for Research Ethics (protocol number 328.159/2013). Each participant provided informed consent.

## 3. Results

### 3.1. Participants Characteristics

Data were analyzed from 9537 drivers and/or motorcyclists who had used alcohol in the previous 30 days (15.8% of the total NHS sample). Supplementary Table 1 (Table S1) presents the characteristics of the study participants by macroregion. Statistical differences among macroregions were found for sex, age, race/skin color, binge drinking, tobacco use, and age at the start of alcohol use (*p* < 0.05).

### 3.2. Prevalence of Driving Under the Influence of Alcohol

**Figure 2:**
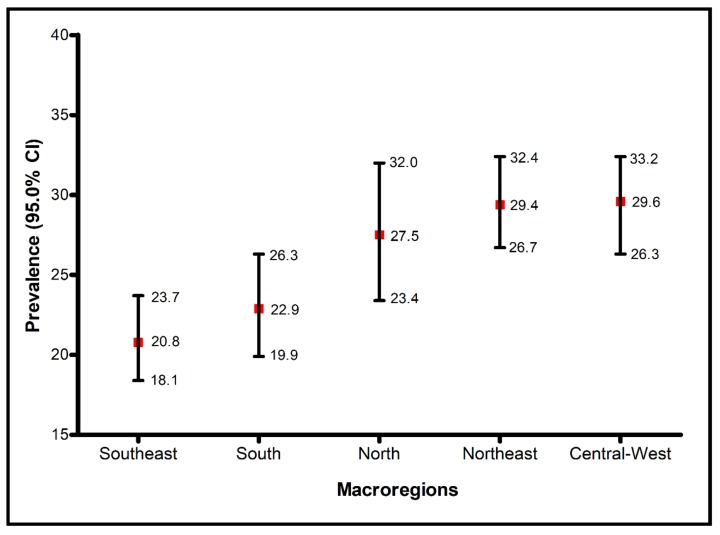
Prevalence of Driving Under the Influence of Alcohol in Brazil, according to macroregion. National Health Survey, 2013.

The prevalence of DUIA varied from 20.8% in the Southeast macroregion to 29.6% in the Central-West (Figure 2). The total prevalence of Brazil was estimated to be 24.3% (95.0% CI: 22.7–26.1).

### 3.3. Predictors of Driving Under the Influence of Alcohol

#### 3.3.1. All Brazil

Table 1 presents the results for DUIA predictors for all Brazil. The pooled analysis revealed associations between DUIA and male sex; age between 30–59 years; education level of high school completion or college school incomplete and college school complete or above; marital status without partner; binge drinking in the previous 30 days; depression; living in outside the capital or metropolitan regions (other regions); and living in the Central-West or Northeast macroregions.

#### 3.3.2. Macroregions

Table 2 shows the results for the Southeast macroregion. Poisson modeling showed associations between DUIA and male sex; age between of 30–39 years; race/skin color black or brown; education level of high school completion or college school incomplete or college school complete or above; depression; binge drinking in the previous 30 days; living outside the capital or metropolitan regions (other regions); and living in the state of Minas Gerais.

Table 3 shows the results for the South macroregion. Multivariable analysis revealed associations between DUIA and male sex; age of 30–39 years; binge drinking; and living outside the capital or metropolitan regions (other regions).

Table 4 shows the results for the Central-West macroregion. Poisson modeling showed associations between DUIA and male sex; education level of high school completion or college school incomplete or college school complete or above; marital status without a partner; binge drinking in the previous 30 days; and living in the Goiás or Mato Grosso states.

Table 5 shows the results for the Northeast macroregion. The analysis showed associations between DUIA and male sex; race/skin color brown as protective factor; education level of high school completion or college school incomplete or college school complete or above; depression; binge drinking in the previous 30 days; living outside the capital or metropolitan regions (other regions); and living in the Maranhão, Piauí, or Rio Grande do Norte states.

Table 6 shows the results for the North macroregion. Poisson regression revealed associations between DUIA and race/skin color brown; education level of elementary school complete or high school incomplete as a protective factor; and living outside the capital or metropolitan regions (other regions).

Table 7 summarize the subpopulations and variables associated with DUIA in Brazil and by macroregion.

## 4. Discussion

This study’s results showed a high prevalence of and some regional disparities in DUIA in Brazil. The estimated prevalence was higher in the Central-West and Northeast macroregions than the Southeast. In addition, the North macroregion also had a high prevalence. The study also identified factors associated with DUIA in each of the country’s macroregions. In most macroregions, the main predictors of DUIA were male sex, high educational level, living in outside the capital or metropolitan regions (other regions) and, binge drinking in the previous 30 days. Depression was also identified as a predictor of DUIA in Brazil and two macroregions.

The prevalence of DUIA found in previous studies varies according to geographical location, methodological differences, economic factors, and sociodemographic or behavioral characteristics of the drivers assessed [21,23,36,37,38]. It is also influenced by country-level differences in legal BAC limits, enforcement, and alcohol use norms [23]. This study found a prevalence of 24.3% (95.0% CI: 22.7–26.1) in Brazil, higher than that of more developed countries. For example, a study conducted in the USA estimated a prevalence of 11.1% [39]. In Spain, a telephone survey of drivers estimated a prevalence of 9.7% [40]. Although comparative analyzes should be performed between developed and developing countries, the hypothesis reported in the literature is that these differences reflect the more rigorous legislation and consequences for DUIA in developed countries compared to those in developing countries, such as Brazil [23]. Considering the 95.0% CI, the prevalence found in this investigation was similar to the results found for the Brazilian population by the Vigitel study in 2013 (26.3%; 95.0% CI: 24.5–28.1) [23].

In this study, the prevalence of DUIA was significantly higher in the Central-West and Northeast macroregions than in the Southeast. The North macroregion also had a high prevalence. The Vigitel study showed a similar distribution among macroregions, with the lowest prevalence of DUIA in the Southeast macroregion (21.7%) and the highest in the Central-West macroregion (33.7%). Prevalence rates estimated for the other macroregions were 29.1% (Northeast), 29.9% (North), and 27.85% (South) [23]. As previously pointed out, economic factors, behavioral and sociodemographic differences between drivers of macroregions, in addition to law enforcement, may explain these differences. Although the absence of an explanatory variable in the Poisson model that indicates the strength of the application of zero tolerance laws in Brazil (for example: Annual percentage of conductors tested in the breathalyzer test or annual number and/or periodicity of sobriety checkpoints performed) or data in the literature that indicate the level of enforcement in each macroregion, the main explanation for the results of this study is differences in the intensity of enforcement and laws between the macroregions [23]. In addition, one possible explanation for these differences is that the Southeast macroregion implemented transit legislation before the enactment of the Brazilian Traffic Code and gave a greater focus on the two main risk factors of RTIs: DUIA and excessive speed [3,41]. Lastly, it is also possible that differences in economic, sociodemographic and behavioral characteristics among macroregions, in addition to increased traffic control in the Southeast and South [23], contributed to the observed disparities. For example, the Southeast macroregion is the richest in Brazil, with the highest GDP, while the Midwest, Northeast and North macroregions are the least developed and with the lowest GDP, which may affect actions to decrease the prevalence of DUIA, such as enforcement intensity, traffic education and other cross-sectional actions. [28]. We also found differences in the prevalence of binge drinking among macroregions, the main risk factor for DUIA [17,20,38,42,43]. The data show a higher prevalence of this behavior in the Northeast (64.4%), Central-West (59.0%) and North (66.2%) macroregions when compared to the Southeast (47.1%) and South (37.4%) (Table S1), which may have influenced the DUIA differences.

The varying prevalence of DUIA may contribute to differences in RTIs among the macroregions of Brazil. In fact, investigations have shown wide variation in these indicators across the country. The NHS highlighted considerable regional differences in the prevalence of RTIs with a rate of 2.4% in the Southeast, 2.9% in the South, 4.4% in the Central-West, 3.4% in the Northeast, and 4.8% in the North macroregions [30]. Another study that investigated rates of hospitalization for RTIs in Brazil found the highest rates in the Central-West (96.6 per 100,000 inhabitants) and Northeast (90.2 per 100,000 inhabitants) macroregions. Rates in the other macroregions were 69.4 (North), 69.8 (South), and 88.6 (Southeast) per 100,000 inhabitants [3]. A study using data from the Mortality Information System found that in 2013 RTIs-related mortality rates in each macroregion were 30.0 (Central-West), 23.5 (Northeast), 23.4 (South), 22.4 (North), and 26.8 (Southeast) per 100,000 [4]. In this study, the prevalence of self-reported RTIs within the previous 12 months was lowest in the Southeast (5.1%; 95.0% CI: 3.9–6.6) and South (5.6%; 95.0% CI: 4.2–7.6) macroregions, and higher in the Central-West (7.4%; 95.0% CI: 5.5–9.9), Northeast (8.2%; 95.0% CI, 6.5–10.1), and North (11.2%; 95.0% CI: 8.4–14.8) (data not shown in tables).

The present study’s results indicated an association between DUIA and an age of 30–39 years in all Brazil and in the Southeast and South macroregions, comparison to drivers aged 18–29 years. These results differ from the majority of studies conducted in more developed countries, which have shown a greater likelihood of this behavior in young adults [39,44]. However, investigations conducted in Brazil have shown a tendency consistent with the present study’s results, which indicate a greater likelihood of DUIA in drivers aged 30 years and older [21,45]. This may reflect the lesser availability of automobiles to younger drivers in Brazil and some macroregions [26]. In the Central-West, Northeast and North macroregions, there was no statistical difference between age groups in relation to the prevalence of DUIA, suggesting greater exposure for this risk factor in all age groups.

Male sex was found to be a risk factor for DUIA in all Brazil and in all macroregions except the North. This is consistent with studies conducted in both developed and developing countries, including Brazil, and provides further evidence for sex differences in the prevalence of DUIA [17,20,23,39,46,47]. Studies show that men are more likely than women to experience RTIs, to die from RTIs, and to engage in risky behaviors, such as DUIA [19,46]. Behavioral, cultural, and gender-related factors explain this association [20]. First, men have a higher prevalence of regular alcohol use and binge drinking [47], which are strong determinants of DUIA. Second, the perception of accident risk tends to be higher in women than men, which may reduce the rate of DUIA in female drivers [20]. Third, women tend to be more cautious about DUIA than men [46]. Finally, gender roles, such as a desire to establish masculinity, increased aggressiveness, demonstration of power, and social status, lead men to engage in more risky behaviors [48], including DUIA.

Differences in the association between DUIA and race/skin color were also observed between macroregions: DUIA was associated with black race/skin color in the Southeast and with brown race/skin color in the North and Southeast. In contrast, brown race/skin color was a protective factor in the Northeast. These results indicate ethnic disparities in the prevalence of DUIA among macroregions. Differences in prevalence between race/color categories can be explained by several variables, such as education and income, acculturation, poor knowledge about the risks of DUIA, misperceptions of risk, misinterpretation of laws and traffic, and related behaviors, which may contribute to mediate this relationship [37]. Our hypothesis for these differences is that enforcement actions in the macroregion may change the focus of the public depending on the macroregion. The Southeast and North macroregions may focus enforcement actions on white individuals, who are the most educated, which may contribute to the higher prevalence of DUIA in blacks and browns in these macroregions; while the Northeast has focused its inspections on individuals of brown race/skin color. However, further study is needed to verify the mechanisms that underlie these variations.

A higher level of education was found to predict DUIA in all Brazil and in the Southeast, Central-West, and Northeast macroregions. The Vigitel study found a similar pattern whereby the frequency of DUIA increased from 2.3% in individuals with illiterate or elementary school incomplete to 9.3% in drivers with complete higher education [23]. Regarding the association between DUIA and high education in Brazil and in two macroregions, individuals with this characteristic generally have more access to vehicles, increasing the likelihood of engaging in risky behaviors, such as DUIA [37]. Also, a possible explanation for this association is that higher levels of schooling have the highest comparative rate of alcohol abuse and problems related to alcohol use [49]. Elementary education was found to be a protective factor in the North macroregion, suggesting a higher prevalence in drivers with low educational level in this macroregion. In the South, differences in the prevalence of DUIA were not observed between levels of education, suggesting that enforcement should focus on drivers of all educational levels.

Some studies have shown that single people adopt more risky behaviors (e.g., excessive speed and DUIA) than those who are married [18,50,51]. Similarly, this study found unmarried status to be a risk factor for DUIA in all Brazil and in the Central-West macroregion. This highlights the increased risk of unmarried individuals of involvement in RTIs, both in Brazil [30], and in other countries [52]. Evidence shows that marriage acts as a protective factor for risky behaviors, such as alcohol use and DUIA, through the partner’s indirect regulation, as well as their social and emotional influence and control [53]. Unmarried (single, widowed, or divorced) individuals tend to have less social support and social control, which may contribute to heavy alcohol use and other high-risk behaviors [54].

Residence outside the capital or metropolitan regions (other regions) was associated with a significantly higher prevalence of DUIA in all Brazil and in the Southeast, South, Northeast, and North macroregions. This may reflect a higher level of laws enforcement in the capitals (e.g., implementation of checkpoints) compared to other regions [26], and suggests the need for interventions to increase oversight in such regions. The absence of statistical difference in the Central-West macroregion may suggest laws enforcement in all regions of residence [26].

Regular alcohol use and binge drinking are associated with physical, psychological, and social consequences, such as chemical dependence, morbidity and mortality, liver cirrhosis, pancreatitis, cancer, infectious diseases, cardiovascular diseases, violence, and RTIs [55]. Consistent with investigations conducted in developed and developing countries [15,21,50], this study found that binge drinking was positively associated with DUIA in all Brazil and in all macroregions except the North, confirming the variable as a predictor of DUIA in drivers. This indicates that health education and traffic-accident prevention should be reinforced for drivers who binge drink.

An association between depression and DUIA was found in all Brazil and in the Southeast and Northeast macroregions. This study is one of the few national studies to consider the depression as a predictor of DUIA in Brazilian drivers. A road survey conducted in Brazil found a high prevalence (19.4%) of depression among drivers with positive BAC [56]. A cross-sectional telephone study of 3979 adults in Ontario (Canada) showed that the probability of DUIA within the previous 12 months increased significantly with an increase in the depressed mood [57]. Some mechanisms may explain this relationship. In general, individuals with depression and/or severe symptoms of depression have a higher frequency of alcohol use, binge drinking, alcohol abuse, and alcohol dependence [58,59]; these are mediators for risky behaviors, such as DUIA, and individuals with depression often use alcohol to self-medicate [60,61]. Additionally, due to diminished social interactions, depressed individuals may be reluctant to seek help from sober drivers after drinking alcohol, which increases their likelihood of DUIA [60]. Finally, depression leads to decreased self-control and willpower, which increases the prevalence of risky behaviors, such as DUIA [60]. The association between depression and DUIA is potentially dangerous, as both factors increase the risk of involvement in RTIs [62]. Quantitative-qualitative investigations are needed to verify the reasons for the higher prevalence of DUIA observed among individuals with depression in the Brazilian context.

The present study has some limitations. First, the cross-sectional design does not allow for determination of cause and effect relationships between DUIA and the associated variables [26]. Second, data on DUIA were self-reported and are, therefore, subject to memory and social desirability response bias [15,26]. Studies that use self-report measures to assess driving under the influence of alcohol may underestimate the true prevalence of this behavior [26,50]. Third, some other variables potentially associated with DUIA (e.g., illicit drug use, psychiatric comorbidities, alcohol dependence, and driving at excessive speed) were not considered. Finally, drivers’ perceptions of and motives for DUIA were not investigated; such data could contribute to a better understanding of the phenomenon.

## 5. Conclusions

Although advances in Brazil’s control of risk factors for RTIs, such as zero tolerance laws for DUIA, the prevalence of DUIA in the country remains high, especially in the Central-West, Northeast and North macroregions. The main factors associated with DUIA in most macroregions were male sex, high educational level, living in outside the capital or metropolitan regions (other regions), and binge drinking in the previous 30 days. Depression has been associated with DUIA in Brazil and in two macroregions.

It is necessary to strengthen traffic safety actions to reduce the magnitude of DUIA in Brazil, such as: (i) Intensifying and increasing the enforcement of zero tolerance laws, with sobriety checkpoints, especially in the larger macroregions prevalence; (ii) focus intensification on the most vulnerable audiences, such as men, with a high educational level and living outside the capitals; (iii) plan interventions according to risk factors for each macroregion; (iv) carry out traffic health education actions aimed at the general population and higher risk subgroups with a focus on reducing the prevalence of DUIA, and (x) carry out health education actions for the general population and drivers in order to reduce the prevalence of alcohol consumption and binge drinking. In addition, cross-sectional interventions are required to reduce the overall prevalence of alcohol use in Brazil. In particular, the intensification of the other strategic actions of the SAFER (Strengthen, Advance, Facilitate, Enforce and Raise) initiative of WHO, which include tightening restrictions on alcohol availability; facilitate access to screening, brief interventions and treatment; enforce comprehensive prohibitions or restrictions on advertising, sponsorship and promotion of alcoholic beverages and raise alcohol prices through taxes and other pricing policies [63]. Also, treatment of depression and mental disorders related to alcohol use in drivers may also contribute to reducing DUIA [60]. Finally, further research is necessary to examine other determinants of DUIA in Brazil, along with qualitative investigations to deepen understanding of why this behavior is adopted by Brazilian drivers.

## Figures and Tables

**Table 1:** Prevalence and Factors Associated with Driving Under the Influence of Alcohol in Brazil. National Health Survey, 2013.

Variables	Total n = 9537	DUIA Prevalence		Bivariate Analysis			Multivariable Analysis		
		%	95.0% CI	cPR	95.0% CI	*p*-Value	aPR	95.0% CI	*p*-Value
Sex									
Female	2146	40.2	36.6–43.9	1.00			1.00		
Male	7391	54.7	51.4–56.0	2.30	1.91–2.77	<0.001	2.38	1.94–2.92	<0.001
Age (years)									
18–29	2697	25.0	22.3–28.0	1.00			1.00		
30–39	2850	28.3	25.3–31.5	1.13	0.97–1.32	0.120	1.21	1.04–1.41	0.014
40–59	3220	22.5	20.0–25.1	0.90	0.76–1.06	0.196	1.09	0.92–1.28	0.309
≥60	770	16.1	12.5–20.6	0.64	0.50–0.84	0.001	0.94	0.72–1.22	0.636
Race/skin color									
White	4575	22.2	19.9–24.7	1.00			1.00		
Black	784	26.3	21.1–32.3	1.19	0.92–1.53	0.181	1.12	0.88–1.43	0.357
Brown	4042	27.5	25.0–30.0	1.24	1.08–1.42	0.002	1.13	0.97–1.31	0.106
Others	136	15.8	9.4–25.3	0.71	0.43–1.19	0.192	0.66	0.39–1.13	0.133
Education									
Illiterate or elementary school incomplete	2387	22.5	19.7–25.5	1.00			1.00		
Elementary school complete or high school incomplete	1434	20.7	17.5–24.3	0.92	0.75–1.12	0.418	0.98	0.80–1.21	0.876
High school complete or college school incomplete	3530	27.9	25.4–30.5	1.24	1.08–1.43	0.002	1.48	1.28–1.70	<0.001
College school complete or above	2186	22.9	20.0–26.0	1.02	0.85–1.21	0.850	1.47	1.21–1.77	<0.001
Marital status									
With partner	5744	22.9	20.8–25.1	1.00			1.00		
Without partner	3793	26.7	24.3–29.3	1.17	1.03–1.33	0.019	1.15	1.01–1.30	0.033
Depression									
No	9024	24.1	22.4–25.8	1.00			1.00		
Yes	513	30.0	23.5–37.4	1.25	0.99–1.57	0.063	1.45	1.15–1.83	0.002
Binge drinking									
No	4312	16.0	14.1–18.1	1.00			1.00		
Yes	5225	32.3	30.2–34.5	2.02	1.78–2.29	<0.001	1.80	1.58–2.05	<0.001
Age at start of alcohol use (years)									
≥18	5002	21.8	19.9–23.9	1.00			1.00		
<18	4535	27.1	24.9–39.4	1.24	1.11–1.38	<0.001	1.06	0.95–1.18	0.325
Tobacco use									
No	7325	24.3	22.5–26.2	1.00					
Yes	2212	24.5	21.5–27.8	1.01	0.88–0.16	0.879			
Residence area									
Capital	4598	21.3	18.9–23.9	1.00			1.00		
Metropolitan region	1279	19.2	16.3–22.5	0.90	0.74–1.10	0.300	0.93	0.77–1.13	0.468
Other regions	3660	26.6	24.3–29.0	1.25	1.08–1.45	0.003	1.32	1.17–1.48	<0.001
Macroregion									
Southeast	2226	20.8	18.1–23.7	1.00			1.00		
South	1648	22.9	19.9–26.3	1.10	0.91–1.34	0.328	1.19	0.98–1.45	0.076
Central-West	1512	29.6	26.3–33.2	1.43	1.19–1.70	<0.001	1.27	1.15–1.63	<0.001
Northeast	2573	29.4	26.7–32.4	1.42	1.20–1.67	<0.001	1.27	1.08–1.50	0.005
North	1578	27.5	23.4–32.0	1.32	1.08–1.62	0.008	1.14	0.95–1.37	0.161

CI: Confidence interval; cPR: Crude prevalence ratio; aPR: Adjusted prevalence ratio; DUIA: Driving Under the Influence of Alcohol.

**Table 2:** Prevalence and Factors Associated with Driving Under the Influence of Alcohol in Southeast Brazil. National Health Survey, 2013.

Variables	Total n = 2226	DUIA Prevalence		Bivariate Analysis			Multivariable Analysis		
		%	95.0% CI	cPR	95.0% CI	*p*-Value	aPR	95.0% CI	*p*-Value
Sex									
Female	523	11.0	8.2–14.7	1.00			1.00		
Male	1703	23.3	20.4–26.5	2.11	1.53–1.90	<0.001	2.26	1.57–3.23	<0.001
Age (years)									
18–29	495	19.2	14.7–24.6	1.00			1.00		
30–39	623	26.9	21.7–32.8	1.40	0.99–1.98	0.056	1.39	1.00–1.91	0.045
40–59	849	20.6	16.4–24.3	1.04	0.73–1.49	0.805	1.26	0.90–1.76	0.171
≥60	259	12.2	8.0–18.2	0.63	0.41–0.99	0.045	1.01	0.62–1.65	0.948
Race/skin color									
White	1329	18.1	15.0–21.7	1.00			1.00		
Black	177	27.3	18.0–39.1	1.50	0.94–2.40	0.084	1.55	1.00–2.38	0.045
Brown	681	24.9	20.1–30.5	1.37	1.06–1.77	0.015	1.37	1.08–1.74	0.009
Others	39	15.0	6.4–31.4	0.82	0.36–1.90	0.659	0.87	0.37–2.03	0.754
Education									
Illiterate or elementary school incomplete	441	16.5	11.8–22.6	1.00			1.00		
Elementary school complete or high school incomplete	320	17.3	12.4–23.6	1.04	0.69–1.57	0.828	1.11	0.71–1.74	0.633
High school complete or college school incomplete	814	23.7	19.9–27.9	1.43	1.04–1.96	0.026	1.70	1.20–2.39	0.002
College school complete or above	651	21.4	17.1–26.5	1.29	0.88–1.88	0.179	1.81	1.22–2.70	0.003
Marital status									
With partner	1310	18.4	6.0–22.1	1.00			1.00		
Without partner	916	23.8	19.7–284	1.26	0.96–1.65	0.097	1.17	0.91–1.52	0.208
Depression									
No	2114	20.4	17.8–23.2	1.00			1.00		
Yes	112	28.8	17.2–44.1	1.41	0.88–1.25	0.148	1.67	1.03–2.69	0.034
Binge drinking									
No	1151	13.6	11.0–16.8	1.00			1.00		
Yes	1075	28.8	25.0–33.0	2.12	1.67–2.68	<0.001	1.95	1.55–2.43	<0.001
Age at start of alcohol use (years)									
≥18	1285	19.9	16.8–23.5	1.00					
<18	941	21.9	18.3–25.9	1.09	0.88–1.35	0.388			
Tobacco use									
No	1663	20.9	18.1–24.0	1.00					
Yes	563	20.5	15.9–26.0	0.98	0.75–1.27	0.889			
Residence area									
Capital	1028	18.1	15.1–21.5	1.00			1.00		
Metropolitan region	393	17.6	13.0–23.3	0.97	0.71–1.32	0.857	0.92	0.71–1.21	0.588
Other regions	805	22.8	19.2–26.9	1.26	0.97–1.64	0.080	1.22	1.00–1.48	0.045
State									
Espírito Santo	254	17.1	20.4–26.9	1.00			1.00		
São Paulo	948	18.5	16.2–21.0	1.08	0.65–1.77	0.758	1.21	0.82–1.79	0.328
Rio de Janeiro	395	20.8	15.6–27.0	1.21	0.69–2.10	0.494	1.32	0.82–2.11	0.246
Minas Gerais	629	26.6	19.4–35.4	1.55	0.88–2.74	0.126	1.58	1.00–2.48	0.045

CI: Confidence interval; cPR: Crude prevalence ratio; aPR: Adjusted prevalence ratio; DUIA: Driving Under the Influence of Alcohol.

**Table 3:** Prevalence and Factors Associated with Driving Under the Influence of Alcohol in the South Macroregion, Brazil. National Health Survey, 2013.

Variables	Total n = 1648	DUIA Prevalence		Bivariate Analysis			Multivariable Analysis		
		%	95.0% CI	cPR	95.0% CI	*p*-Value	aPR	95.0% CI	*p*-Value
Sex									
Female	443	9.6	6.1–14.9	1.00			1.00		
Male	1205	26.9	23.4–30.7	2.79	1.77–4.38	<0.001	2.56	1.63–4.02	<0.001
Age (years)									
18–29	410	21.7	16.6–27.8	1.00			1.00		
30–39	467	27.7	22.1–34.0	1.27	0.89–1.81	0.172	1.48	1.05–2.06	0.023
40–59	591	21.7	17.1–27.2	1.00	0.67–1.49	0.985	1.20	0.81–1.77	0.354
≥60	180	17.3	10.3–27.5	0.79	0.51–1.24	0.319	1.15	0.77–1.71	0.488
Race/skin color									
White	1410	22.5	19.1–26.5	1.00			1.00		
Black	63	13.7	7.3–24.4	0.60	0.32–1.14	0.125	0.59	0.32–1.11	0.103
Brown	163	28.7	19.4–40.3	1.27	0.83–1.93	0.256	1.07	0.69–1.66	0.732
Others	12	14.4	3.8–41.6	0.64	0.18–2.26	0.488	0.56	0.13–2.40	0.445
Education									
Illiterate or elementary school incomplete	331	21.9	16.0–29.2	1.00					
Elementary school complete or high school incomplete	212	19.6	12.7–29.0	0.89	0.51–1.53	0.684			
High school complete or college school incomplete	593	26.3	21.1–32.2	1.19	0.89–1.61	0.230			
College school complete or above	512	20.6	15.7–26.5	0.93	0.61–1.42	0.767			
Marital status									
With partner	1023	21.8	18.4–25.7	1.00					
Without partner	625	25.0	20.0–30.7	1.14	0.88–1.47	0.302			
Depression									
No	1564	23.0	18.9–26.5	1.00					
Yes	84	20.1	11.9–32.1	0.87	0.52–1.45	0.605			
Binge drinking									
No	1047	16.1	12.9–19.9	1.00			1.00		
Yes	601	34.3	28.7–40.4	2.13	1.65–2.74	<0.001	1.95	1.49–2.55	<0.001
Age at start of alcohol use (years)									
≥18	825	19.9	16.2–24.2	1.00			1.00		
<18	823	25.8	21.7–30.4	1.29	1.02–1.64	0.030	1.08	0.84–1.39	0.521
Tobacco use									
No	1292	22.3	18.9–26.1	1.00					
Yes	356	25.5	19.5–32.5	1.14	0.85–1.51	0.365			
Residence area									
Capital	751	17.4	14.5–20.7	1.00			1.00		
Metropolitan region	307	18.3	13.6–24.1	1.04	0.71–1.53	0.808	1.07	0.76–1.51	0.688
Other regions	590	24.6	20.9–28.8	1.41	1.02–1.95	0.034	1.36	1.01–1.83	0.039
State									
Rio Grande do Sul	664	21.8	16.8–27.7	1.00			1.00		
Paraná	589	23.5	18.8–29.0	1.07	0.77–1.50	0.653	1.09	0.78–1.53	0.598
Santa Catarina	395	24.0	18.1–31.0	1.10	0.76–1.59	0.610	1.05	0.71–1.55	0.801

CI: Confidence interval; cPR: Crude prevalence ratio; aPR: Adjusted prevalence ratio; DUIA: Driving Under the Influence of Alcohol.

**Table 4:** Prevalence and Factors Associated with Driving Under the Influence of Alcohol in the Central-West Macroregion, Brazil. National Health Survey, 2013.

Variables	Total n = 1512	DUIA Prevalence		Bivariate Analysis			Multivariable Analysis		
		%	95.0% CI	cPR	95.0% CI	*p*-Value	aPR	95.0% CI	*p*-Value
Sex									
Female	402	14.7	10.5–20.3	1.00			1.00		
Male	1110	34.1	29.9–38.6	2.31	1.61–3.31	<0.001	2.53	1.72–3.73	<0.001
Age (years)									
18–29	441	32.4	25.7–40.0	1.00			1.00		
30–39	436	31.4	24.3–39.4	0.96	0.72–1.29	0.818	1.12	0.79–1.58	0.510
40–59	537	26.4	22.1–31.1	0.83	0.58–1.12	0.211	1.10	0.80–1.49	0.567
≥60	98	22.4	11.1–39.9	0.68	0.35–1.32	0.264	0.97	0.57–1.63	0.923
Race/skin color									
White	667	28.1	24.3–32.3	1.00			1.00		
Black	100	33.3	23.3–45.0	1.18	0.78–1.78	0.419	1.06	0.71–1.58	0.751
Brown	724	32.1	26.3–36.4	1.10	0.93–1.30	0.230	1.09	0.91–1.30	0.307
Others	21	8.5	1.9–30.2	0.30	0.07–1.26	0.100	0.27	0.05–1.45	0.128
Education									
Illiterate or elementary school incomplete	370	20.7	15.9–26.6	1.00			1.00		
Elementary school complete or high school incomplete	233	29.9	20.8–41.0	1.44	0.86–2.42	0.163	1.50	0.91–2.45	0.106
High school complete or college school incomplete	552	34.0	28.1–40.4	1.64	1.15–2.32	0.006	1.83	1.32–2.51	<0.001
College school complete or above	357	32.0	26.4–38.3	1.54	1.13–2.11	0.006	2.12	1.53–2.95	<0.001
Marital status									
With partner	888	26.7	23.9–29.6	1.00			1.00		
Without partner	624	34.4	27.7–41.7	1.28	1.03–1.60	0.024	1.21	1.00–1.46	0.049
Depression									
No	1429	29.1	25.6–32.8	1.00			1.00		
Yes	83	38.2	27.4–50.4	1.32	0.94–1.83	0.109	1.38	0.97–1.95	0.066
Binge drinking									
No	652	19.3	14.2–25.5	1.00			1.00		
Yes	860	36.9	33.4–40.4	1.91	1.44–2.54	<0.001	1.74	1.33–2.26	<0.001
Age at start of alcohol use (years)									
≥18	806	24.2	20.1–28.9	1.00			1.00		
<18	706	35.4	29.2–42.1	1.46	1.09–1.95	0.010	1.23	0.93–1.62	0.123
Tobacco use									
No	1158	30.7	26.5–35.1	1.00					
Yes	354	25.8	20.7–31.6	0.73	0.63–1.10	0.210			
Residence area									
Capital	823	28.3	24.7–32.2	1.00					
Metropolitan region	73	29.5	23.8–36.0	1.04	0.81–1.33	0.741			
Other regions	616	30.6	25.1–36.8	1.08	0.85–1.36	0.512			
State									
Distrito Federal	388	24.7	21.1–28.7	1.00			1.00		
Mato Grosso do Sul	403	26.3	13.4–29.4	1.06	0.87–1.28	0.533	1.11	0.95–1.30	0.166
Mato Grosso	273	32.9	24.8–42.1	1.32	0.98–1.80	0.070	1.43	1.08–1.88	0.011
Goiás	448	32.7	26.0–38.1	1.28	1.00–1.64	0.047	1.40	1.15–1.70	0.001

CI: Confidence interval; cPR: Crude prevalence ratio; aPR: Adjusted prevalence ratio; DUIA: Driving Under the Influence of Alcohol.

**Table 5:** Prevalence and Factors Associated with Driving Under the Influence of Alcohol in the Northeast Macroregion, Brazil. National Health Survey, 2013.

Variables	Total n = 2573	DUIA Prevalence		Bivariate Analysis			Multivariable Analysis		
		%	95.0% CI	cPR	95.0% CI	*p*-Value	aPR	95.0% CI	*p*-Value
Sex									
Female	445	13.0	9.1–18.2	1.00			1.00		
Male	2128	32.2	29.1–35.5	2.47	1.73–3.53	<0.001	2.54	1.75–3.69	<0.001
Age (years)									
18–29	816	30.6	15.8–35.8	1.00			1.00		
30–39	805	30.2	25.2–35.7	0.98	0.78–1.24	0.913	1.03	0.82–1.29	0.772
40–59	780	27.8	24.1–31.8	0.90	0.73–1.12	0.367	0.98	0.76–1.27	0.934
≥ 60	172	26.1	16.0–39.7	0.85	0.52–1.39	0.528	0.99	0.61–1.61	0.980
Race/skin color									
White	779	33.9	27.6–40.9	1.00			1.00		
Black	291	26.5	19.3–35.2	0.78	0.55–1.10	0.161	0.79	0.57–1.10	0.174
Brown	1473	28.0	24.5–31.8	0.82	0.64–1.05	0.131	0.77	0.61–0.98	0.040
Others	30	12.5	3.7–34.8	0.36	0.11–1.18	0.093	0.44	0.13–1.45	0.178
Education									
Illiterate or elementary school incomplete	850	27.9	23.4–32.8	1.00			1.00		
Elementary school complete or high school incomplete	381	25.5	19.3–33.0	0.91	0.67–1.23	0.566	0.91	0.67–1.23	0.557
High school complete or college school incomplete	931	34.1	29.4–39.1	1.22	0.96–1.54	0.091	1.41	1.12–1.77	0.003
College school complete or above	411	26.4	20.5–33.3	0.94	0.72–1.24	0.699	1.33	1.00–1.76	0.044
Marital status									
With partner	1596	29.3	25.9–32.8	1.00					
Without partner	977	29.7	24.6–35.4	1.01	0.80–1.27	0.901			
Depression									
No	2429	29.1	26.3–32.0	1.00			1.00		
Yes	144	36.8	26.0–49.1	1.26	0.91–1.74	0.150	1.51	1.10–2.07	0.011
Binge drinking									
No	915	19.8	15.5–24.8	1.00			1.00		
Yes	1658	35.0	31.6–38.6	1.77	1.38–2.27	<0.001	1.67	1.29–2.14	<0.001
Age at start of alcohol use (years)									
≥ 18	1258	26.0	22.1–30.3	1.00			1.00		
< 18	1315	32.7	29.3–36.3	1.25	1.04–1.50	0.014	1.15	0.95–1.39	0.145
Tobacco use									
No	2025	29.1	25.7–32.6	1.00					
Yes	548	30.7	24.7–37.4	1.05	0.81–1.26	0.671			
Residence area									
Capital	1145	25.6	21.0–30.7	1.00			1.00		
Metropolitan region	388	21.3	15.3–29.0	0.83	0.57–1.21	0.342	0.86	0.60–1.23	0.410
Other regions	1040	32.4	19.0–36.0	1.26	1.01–1.57	0.035	1.34	1.08–1.66	0.006
State									
Alagoas	205	20.5	13.3–30.2	1.00			1.00		
Maranhão	215	38.9	30.4–48.2	1.90	1.18–3.04	0.008	1.96	1.27–3.02	0.002
Piauí	319	37.1	30.9–43.7	1.81	1.15–2.83	0.009	1.89	1.23–2.91	0.004
Ceará	313	30.8	24.0–38.3	1.49	0.93–2.40	0.093	1.52	0.96–2.39	0.068
Rio Grande do Norte	265	36.1	26.7–46.7	1.76	1.07–2.90	0.026	1.67	1.10–2.52	0.015
Paraíba	228	32.9	23.0–44.6	1.60	0.94–2.72	0.079	1.59	0.99–2.55	0.052
Pernambuco	358	22.5	15.7–32.1	1.09	0.64–1.88	0.732	1.08	0.66–1.75	0.751
Sergipe	235	24.8	21.4–28.5	1.21	0.78–1.87	0.390	1.30	0.86–1.98	0.207
Bahia	435	27.3	22.7–32.5	1.33	0.85–2.09	0.207	1.29	0.85–1.96	0.230

CI: Confidence interval; cPR: Crude prevalence ratio; aPR: Adjusted prevalence ratio; DUIA: Driving Under the Influence of Alcohol.

**Table 6:** Prevalence and Factors Associated with Driving Under the Influence of Alcohol in the North Macroregion, Brazil. National Health Survey, 2013.

Variables	Total n = 1578	DUIA Prevalence		Bivariate Analysis			Multivariable Analysis		
		%	95.0% CI	cPR	95.0% CI	*p*-Value	aPR	95.0% CI	*p*-Value
Sex									
Female	333	21.1	11.9–34.5	1.00			1.00		
Male	1245	28.8	24.8–33.2	1.36	0.81–2.30	0.239	1.43	0.89–2.30	0.138
Age (years)									
18–29	535	32.8	24.5–42.2	1.00			1.00		
30–39	519	27.8	21.2–35.6	0.84	0.58–1.22	0.383	0.89	0.63–1.27	0.554
40–59	463	20.3	14.0–28.4	0.61	0.37–1.02	0.060	0.72	0.43–1.21	0.221
≥ 60	61	13.9	6.5–27.1	0.42	0.19–0.92	0.032	0.59	0.24–1.44	0.251
Race/skin color									
White	390	21.6	16.5–27.7	1.00			1.00		
Black	153	32.8	21.7–43.9	1.47	0.91–2.38	0.113	1.31	0.81–2.12	0.265
Brown	1001	28.9	23.2–35.1	1.33	0.98–1.81	0.066	1.32	1.03–1.69	0.026
Others	34	36.7	14.9–65.7	1.70	0.72–3.99	0.223	1.47	0.74–2.90	0.262
Education									
Illiterate or elementary school incomplete	395	28.6	21.6–36.7	1.00			1.00		
Elementary school complete or high school incomplete	288	16.0	11.3–22.3	0.56		0.015	0.52	0.32–0.85	0.010
High school complete or college school incomplete	640	33.7	27.2–40.9	1.17	0.35–0.89	0.229	1.22	0.88–1.70	0.216
College school complete or above	255	21.0	15.6–27.7	0.73	0.90–1.54	0.156	0.95	0.61–1.48	0.848
Marital status					0.47–1.12				
With partner	927	24.7	20.3–29.6	1.00			1.00		
Without partner	651	32.6	25.1–38.8	1.27	0.97–1.67	0.074	1.27	0.97–1.67	0.076
Depression									
No	1488	27.5	23.4–32.1	1.00					
Yes	90	26.4	12.4–47.6	0.95	0.47–1.92	0.902			
Binge drinking									
No	547	21.8	15.6–29.7	1.00			1.00		
Yes	1031	30.4	25.5–35.8	1.38	0.97–1.97	0.067	1.23	0.85–1.77	0.264
Age at start of alcohol use (years)									
≥18	828	24.3	20.0–29.2	1.00			1.00		
<18	750	31.2	24.0–39.4	1.28	0.93–1.75	0.117	1.17	0.88–1.55	0.258
Tobacco use									
No	1187	27.0	22.7–31.9	1.00					
Yes	391	29.2	23.1–36.1	1.08	0.85–1.35	0.510			
Residence area									
Capital	851	19.6	15.5–24.6	1.00			1.00		
Metropolitan region	118	22.6	18.4–27.5	1.15	0.84–1.56	0.357	1.20	0.87–1.66	0.246
Other regions	609	32.1	27.1–37.6	1.63	1.23–2.17	0.001	1.74	1.33–2.26	<0.001
State									
Alagoas	242	24.4	18.1–32.1	1.00			1.00		
Maranhão	204	26.4	21.7–31.7	1.07	0.76–1.52	0.662	0.88	0.65–1.19	0.419
Piauí	172	25.1	18.1–33.7	1.02	0.67–1.57	0.900	0.98	0.77–1.25	0.913
Ceará	280	35.2	29.1–41.9	1.44	1.02–2.02	0.035	1.50	0.94–2.39	0.085
Rio Grande do Norte	227	27.9	20.2–37.1	1.14	0.75–1.74	0.537	0.88	0.64–1.23	0.484
Paraíba	148	26.4	22.1–31.1	1.07	0.77–1.50	0.655	1.18	0.85–1.63	0.302
Pernambuco	305	30.0	23.5–37.3	1.22	0.84–1.77	0.277	1.06	0.75–1.50	0.707
Sergipe	235	24.8	21.4–28.5	1.21	0.78–1.87	0.390	1.30	0.86–1.98	0.207
Bahia	435	27.3	22.7–32.5	1.33	0.85–2.09	0.207	1.29	0.85–1.96	0.230

CI: Confidence interval; cPR: Crude prevalence ratio; aPR: Adjusted prevalence ratio; DUIA: Driving Under the Influence of Alcohol.

**Table 7:** Subpopulations and Variables Associated with Driving Under the Influence of Alcohol in Brazil and Macroregions. National Health Survey, 2013.

Variables	Brazil	Southeast	South	Central-West	Northeast	North
Age (years)	30–39	30–39	30–39	-	-	-
Sex	Male	Male	Male	Male	Male	-
Race/skin color	-	Black and Brown	-	-	Brown*	Brown
Education	Elementary school complete or high school incomplete and College school complete or above	Elementary school complete or high school incomplete and College school complete or above	-	Elementary school complete or high school incomplete and College school complete or above	Elementary school complete or high school incomplete and College school complete or above	Elementary school complete or high school incomplete**
Marital status	Without partner	-	-	Without partner	-	-
Binge drinking	Yes	Yes	Yes	Yes	Yes	-
Depression	Yes	Yes	-	-	Yes	-
Residence area	Living in outside the capital or metropolitan regions (other regions)	Living in outside the capital or metropolitan regions (other regions)	Living in outside the capital or metropolitan regions (other regions)	-	Living in outside the capital or metropolitan regions (other regions)	Living in outside the capital or metropolitan regions (other regions)
State	-	Minas Gerais	-	Goias and Mato Grosso	Maranhão, Piauí and Rio Grande do Norte	-
Macroregion	Central-West and Northeast	-	-	-	-	-

* Protection factor when compared to white color; ** Protection factor when compared to educational level Illiterate or elementary school incomplete.

## Supplementary Materials

The following are available online at https://www.mdpi.com/1660-4601/17/3/767/s1. Table S1: Descriptive Analysis of Sociodemographic and Behavioral Characteristics in the Sample Study, by Macro–region of Brazil. National Health Survey, 2013
