# Supplementary material for: Prevalence and Factors Associated with Driving Under the Influence of Alcohol in Brazil: An Analysis by Macroregion
Source: Int J Environ Res Public Health. 2020 Jan 25;17(3):767. doi: 10.3390/ijerph17030767 (PMC7037342; doi:10.3390/ijerph17030767)
Supplement: Supplementary file 1 [file ijerph-17-00767-s001.pdf]

Table S1. Descriptive Analysis of Sociodemographic and Behavioral Characteristics in the Sample Study, by Macroregion of Brazil. National Health Survey, 2013.

Variables	Total (n = 9537)	Southeast (n = 2226)	South (n = 1648)	Central–West (n = 1512)	Northeast (n = 2573)	North (n = 1578)	□2	p–value*
Gender								
Female	19.7 (18.1–21.5)	20.6 (17.6–20.1)	20.1 (19.7–26.8)	23.1 (20.4–26.1)	14.6 (12.7–16.8)	17.1 (13.8–21.1)	5.97	0.005
Male	80.3 (78.5–81.9)	79.4 (75.9–82.4)	76.9 (73.2–80.3)	76.9 (74.0–79.6)	85.4 (83.2–87.3)	82.9 (78.9–86.2)		
Age (years)								
18–29	31.5 (29.7–33.3)	26.8 (24.0–29.8)	30.8 (27.3–40.2)	35.9 (31.8–40.2)	36.8 (33.5–40.2)	41.3 (36.8–46.1)	7.60	< 0.001
30–39	27.3 (25.8–29.0)	26.9 (24.0–29.9)	26.3 (23.8–29.1)	26.3 (23.8–29.1)	29.5 (26.7–32.3)	29.3 (25.4–33.6)		
40–59	32.5 (30.8–34.3)	35.0 (31.8–38.2)	31.8 (28.6–35.2)	31.8 (28.6–35.2)	27.6 (25.4–39.9)	26.7 (23,4–30.3)		
≥ 60	8.6 (7.3–10.0)	11.3 (9.1–14.0)	6.0 (4.5–7.8)	6.0 (4.6–7.8)	6.1 (4.8–7.9)	2.6 (1.6–4.2)		
Race/skin color								
White	55.3 (51.8–58.7)	62.2 (57.2–66.9)	84.1 (79.5–87.9)	42.8 (39.9–46.9)	29.8 (26.5–33.3)	23.6 (19.2–28.7)	52.94	< 0.001
Black	7.7 (6.7–8.9)	7.8 (6.1–9.8)	3.8 (2.4–6.2)	5.6 (4.4–7.1)	11.5 (9.0–14.6)	8.1 (6.6–10.1)		
Brown	36.0 (33.0–39.0)	28.8 (24.8–33.2)	11.6 (8.9–15.0)	50.1 (46.0–54.2)	57.6 (54.3–60.8)	66.3 (60.7–71.5)		
Others	1.1 (0.8–1.4)	1.2 (0.7–1.9)	0.4 (0.2–0.8)	1.4 (0.7–2.8)	1.0 (0.7–1.6)	1.9 (1.1–3.4)		
Education								
Illiterate or elementary school incomplete	24.9 (22.8–27.2)	18.7 (26.1–21.6)	24.8 (21.0–29.1)	24.8 (19.6–30.9)	36.5 (31.9–41.5)	25.4 (21.3–29.9)	7.47	< 0.001
Elementary school complete or high school incomplete	16.0 (14.6–17.5)	15.7 (13.4–18.8)	15.3 (12.5–18.9)	16.4 (14.0–19.0)	16.1 (13.8–18.7)	18.8 (14.5–24.1)		
High school complete or college school incomplete	38.1 (36.1–40.1)	39.6 (36.2–43.2)	37.6 (34.3–41.0)	38.1 (34.1–42.2)	34.3 (30.1–38.7)	43.3 (37.2–49.6)		
College school complete or above	21.1 (18.5–23.8)	26.0 (21.3–31.2)	22.2 (18.3–26.7)	20.7 (14.7–28.2)	13.1 (10.3–16.5)	12.4 (9.3–16.5)		
Marital status								
With partner	62.3 (60.6–64.0)	60.7 (57.8–63.6)	66.6 (62.7–70.4)	61.9 (58.5–65.1)	62.3 (59.1–65.4)	59.4 (54.6–64.1)	2.45	0.055
Without partner	37.7 (36.0–34.9)	39.3 (36.4–42.2)	33.4 (29.6–37.3)	38.1 (34.9–41.5)	37.7 (34.6–40.9)	40.6 (35.9–45.4)		
Depression								
No	95.4 (94.6–96.0)	95.3 (93.7–96.6)	95.7 (94.0–97.0)	94.0 (92.0–95.5)	95.4 (94.0–96.5)	95.9 (94.2–97.2)	0.54	0.662
Yes	4.6 (3.9–5.4)	4.7 (3.4–6.2)	4.3 (3.0–6.0)	6.0 (4.5–8.0)	4.6 (3.5–5.9)	4.1 (2.8–5.8)		
Binge drinking								
No	48.9 (46.8–51.2)	52.9 (49.6–56.1)	62.6 (58.4–66.7)	41.0 (37.4–44.7)	36.6 (33.0–44.4)	33.8 (30.3–37.5)	37.44	< 0.001

Yes	50.1 (48.8–53.1)	47.1 (43.9–50.3)	37.4 (33.3–41.6)	59.0 (55.3–62.6)	63.4 (59.6–67.0)	66.2 (62.5–69.7)		
Age at start of alcohol use (years)								
≥ 18	52.3 (50.5–54.1)	55.8 (52.6–59.0)	49.2 (45.1–53.2)	51.6 (48.1–55.1)	48.5 (45.3–51.7)	53.4 (48.3–58.4)	4.47	0.003
< 18	47.7 (45.8–49.5)	44.2 (41.0–47.4)	50.8 (46.8–54.9)	48.4 (44.9–51.9)	51.5 (48.3–54.7)	46.6 (41.6–51.7)		
Tobacco use								
No	77.3 (75.6–78.9)	74.8 (71.5–77.9)	82.0 (78.9–84.8)	78.9 (76.2–81.4)	77.1 (74.5–79.6)	78.6 (75.8–81.2)	4.72	0.003
Yes	22.7 (21.0–24.4)	25.2 (22.1–28.5)	18.0 (15.2–21.1)	21.1 (18.6–23.8)	22.9 (20.4–25.5)	21.4 (19.0–24.2)		
Residence area								
Capital	24.8 (19.1–31.5)	25.0 (15.0–38.7)	15.0 (8.1–26.0)	39.1 (23.7–57.0)	25.8 (17.5–36.2)	32.1 (18.5–49.6)	1.29	0.254
Metropolitan region	12.9 (8.5–18.9)	16.8 (8.7–30.0)	10.4 (5.7–21.7)	7.8 (2.3–20.2)	10.9 (5.9–19.2)	6.6 (1.8–21.9)		
Other	62.3 (55.2–69.9)	58.2 (44.6–70.7)	74.6 (61.9–84.2)	53.0 (36.0–69.0)	63.3 (52.8–72.7)	61.3 (43.6–76.4)		

Note: Data are presented as % (95.0%CI); *Pearson's chi–square test.
